# Observation of Direct and Indirect Effects of Surface Stabilizer on the Attenuation Coefficient of CdTe Nanoplatelet Films

**DOI:** 10.3390/nano15221688

**Published:** 2025-11-07

**Authors:** Sergei Bubenov, Aigerim Ospanova, Alexander Vinokurov, Asset Kainarbay, Aizhan Akhmetova, Kirill Cherednichenko, Dulat Daurenbekov, Sergey Dorofeev

**Affiliations:** 1 Department of Chemistry, Lomonosov Moscow State University, Leninskie Gory 1–3, 119991 Moscow, Russia; 2 Institute of Physical and Technical Sciences, L.N. Gumilyov Eurasian National University, Kazhymukan Str. 13, 010000 Astana, Kazakhstan; kainarbai_azh@enu.kz (A.K.);

**Keywords:** nanoplatelets, CdTe, absorption coefficient, ligand exchange, film, total X-ray fluorescence

## Abstract

Absorption spectra are widely used in laboratory practice to measure the content of a great variety of colloidal semiconductor nanocrystals. In the case of atomically thin nanoplatelets, only CdSe has been studied enough to allow such quantification, while CdTe nanoplatelets—a promising material for photodetection—are understudied in this regard. In this work, a powerful combination of total XRF spectroscopy, absorption spectroscopy and profilometry was employed for thin films to extract the absorption coefficient values. The morphology and surface composition of nanoplatelets were studied with TEM and IR spectroscopy. The molar absorption coefficient of oleate-terminated CdTe nanoplatelets at the first optical transition was measured at about 5 × 10^4^ L·mol^−1^·cm^−1^ (per mole of Te), which is among the highest values for AIIBVI nanomaterials. The exchange of stabilizer with hexadecanethiol induced an approximately 5-fold decrease in the volume fraction of semiconductor material in thin films and a 5-fold decrease in absorbance. The latter effect is linked to the formation of a quasi-type II heterojunction between CdTe cores and effectively half of a monolayer shell of CdS. The density effect is explained by the diminished capacity of nanoscrolls for close packing. The combination of XRF and profilometry is proposed as a technique for fast nanomorphology evaluation.

## 1. Introduction

Semiconductor nanomaterials have been extensively researched in recent decades. In large part this is due to their robust and often tunable optical properties. The focal point of this attention has gradually shifted from colloidal AIIBVI quantum dots [1] to the largely lead-based perovskite systems [2] and then to more sophisticated materials like nanoplatelets (NPLs) [3] and colloidal quantum shells [4]. The same optical properties are frequently used as a readily available and quick measure of the mean size of an ensemble, polydispersity [5] or concentration of nanoparticles. It is not surprising, then, that the seminal work of Peng et al. on calibration curves for cadmium chalcogenide quantum dots [6] has accumulated more than 6 thousand citations to date.

NPLs are flexible 2D nanostructures that are essentially monodisperse in their thickness of just several atomic layers, but can be grown to hundreds of nanometers in their lateral dimensions [7,8,9]. Their inorganic cores are almost entirely “surface” in the sense that the whole lattice is distorted and a lowered crystal symmetry can be observed in diffraction patterns [9]. Of course, in these conditions, the surface capping agent plays an integral role in the formation and stabilization of the material and can even impart its own properties, like optical rotation, to the inorganic core [9]. NPLs are also sought after due to their uniquely narrow absorption and photoluminescence features [10] and giant oscillator strengths at low temperatures [11], high exciton-binding energy [12], picosecond radiative recombination and high efficiency [13].

Unexpectedly, the literature corpus on absorption coefficients of atomically thin NPLs consists of only a few well-executed studies. CdSe is, yet again, the more studied system: the spectral dependencies were reported not only on the principal parameter of NPL thickness [14,15,16,17], but also on the lateral size, on which an unusual non-quadratic scaling was observed [14]. The same set of publications also contains theoretical insight into absorption coefficients. As for CdTe NPLs, there is a single work [18] on these objects wherein UV-Vis spectroscopy was combined with optical profilometry to obtain an absorption coefficient α of 1.5 × 10^4^ cm^−1^ for thin films. Cadmium telluride (CdTe) is a direct-band-gap II–VI semiconductor (Eg ≈ 1.5 eV at 300 K) with a high absorption coefficient and mature processing routes, that explain its dominant role in thin-film photovoltaics and other optoelectronic platforms. More recently, CdTe NPLs reached improved emission efficiencies and promising scintillation behavior, enabling detailed photophysics under both optical and ionizing excitation [19,20,21,22,23]. Early CdTe-NPL films already showed measurable photoresponse, highlighting their device relevance in thin-film configurations [18].

NPLs are sometimes viewed as hybrid organo-inorganic systems due to crucial effects of the nature of surface passivation on the optical properties. Soft Pearson acid nature of group XII metal ions ensures a prominent role of thiolate and other sulfur-containing ligands. The mechanism of thiolate ligation in CdTe NPLs is strongly dependent on the polymorphic modification: wurtzite platelets are dominated by neutral L-/Z-types, so they exchange the Cd(SR)_2_ fragment [24], whereas cubic (zinc-blende) plates exhibit pronounced polar cationic facets that prefer anionic X-type ligands [7]. Characteristically, thiol exchange with native carboxylates alters not only the spectra but also the morphology: spontaneous sheetlet folding has been demonstrated for CdTe NPLs after exchange with alkylthiols [7]. For the related case of CdSe NPLs, thiol ligands cause a red shift of the lowest-energy exciton transition; this shift is consistent with the formation of a sulfur layer on the surface, which effectively increases the effective thickness by ~1 monolayer (½ ML of sulfur on each side), as confirmed by comparison with the mass-load model and the Raman signature of CdS SO phonons in the 200–300 cm^−1^ region [25].

The flat two-dimensional shape of CdTe nanosheets enhances their technological compatibility with flexible substrates and typical microfabrication methods, expanding the possibilities of use in flexible electronic and optoelectronic systems. In our work we would like to build upon the existing work on the optical response of CdTe NPL films with addition of another powerful characterization technique—TXRF (total X-ray fluorescence) spectroscopy. In a “bottom-up” approach to our deductions, we aim to explore the relations between the nature of passivating ligand, nanomorphology and light attenuation in a NPL film.

We propose a unified protocol applied to the flat CdTe NPLs specimens and the ribbon-like scrolls obtained after ligand exchange, combining TXRF/XRF (elemental composition and relative film areal density), UV–visible absorption spectroscopy (exciton peak positions/linewidths), TEM (morphology, radius of curvature), and profilometry (thickness/topography). This integrated workflow enables a direct comparison of flat and “scroll” morphologies and allows film thickness and nanomorphology to be evaluated—without mandatory TEM—via the XRF/profilometry pairing.

## 2. Materials and Methods

### 2.1. Chemicals

The chemicals used were 1-octadecene (Sigma-Aldrich, St. Louis, MO, USA, 90%), propionic acid (Sigma-Aldrich, 99%), oleic acid (Sigma-Aldrich, 90%), cadmium acetate dihydrate (Cd(OAc)_2_·2H_2_O) (>98%), tellurium powder (Te) (98%), hexadecanthiol (Aldrich, St. Louis, MO, USA, >95%), oleylamine (Aldrich, 70%), trioctylphosphine (Aldrich, 95%), hexane (>99%), ethanol (95%), acetone (>99%) dichloromethane (CH_2_Cl_2_) (>99%).

### 2.2. Precursor Preparation

Cadmium propionate Cd(Prop)_2_: to prepare the cadmium propionate precursor, 8.07 mmol (2.15 g) of cadmium acetate dihydrate and 10 mL (133 mmol) of propionic acid were mixed in a 50 mL three-neck flask and stirred at 90 °C under argon for one hour. After one hour, the mixture was cooled to approximately 50–60 °C and acetone was introduced until the solution turned turbid. White precipitate of cadmium propionate was filtered and dried.

Trioctyolphosphine telluride: to prepare the tellurium precursor 1 mmol (0.1276 g) of tellurium powder was added to 1 mL of trioctylphosphine. The powder was dispersed with the use of an ultrasonic bath until full tellurium powder dissolution.

### 2.3. Synthesis of CdTe NPLs

In present work we utilized the synthetic technique proposed by Pedetti et al. [18] with our minor modification described in [26]. Briefly, for typical synthesis of CdTe NPLs 0.5 mmol (130 mg) of cadmium propionate, 0.25 mmol (80 μL) of oleic acid and 10 mL of 1-octadecene were added into 50 mL three-neck flask. The mixture was heated under argon to 110 °C with stirring and degassed for 30 min. After that the temperature was raised to 170 °C and 100 μL of 1 M trioctylphosphine telluride solution diluted by 0.5 mL of 1-octadecene were injected. Upon injection, the color changes to dark yellow and then to orange. After 20 min of NPLs growth 1 mL of oleic acid was injected and the mixture was cooled to room temperature. The product was isolated by addition of an excess of acetone (1:2 by volume) with subsequent centrifugation at 8000 rpm for 5 min and ultrasound-assisted redispersion in hexane. The amount of hexane used was roughly 1 mL per 1 mg of NPLs.

### 2.4. Ligand Exchange

2 mL (4.2 μmol in cadmium) of raw CdTe NPLs sample were mixed with 8 mL of hexane and 800 μL of hexadecanethiol and stirred under argon for 30 min for degassing. After 30 min the temperature was raised to 65 °C and 150 μL of oleylamine were injected and the mixture was stirred for 1 h. Upon cooling to room temperature, the sol color remained yellow, while successful ligand exchange should result in red or dark-red color [7]. The mixture was left for 2 days to complete the exchange reaction what resulted in red coloration. Equal volume of acetone was introduced to precipitate the NPLs from the reaction mass. The product was centrifuged at 18,000 rpm for 2 min and redispersed in hexane. The amount of hexane used was roughly 1 mL per 1 mg of NPLs.

### 2.5. Spin-Coating Technique

In order to deposit uniform films, carboxylated CdTe NPLs were redispersed in CH_2_Cl_2_. The particles were deposited from 1 mL of a hexane sol (8000 rpm, 5 min of centrifugation) and then dispersed in 1.5 mL of CH_2_Cl_2_ with sonication. Thiolated CdTe NPLs sample dispersed in CH_2_Cl_2_ gave non-uniform films so we used initial hexane-based sols. The films were formed by deposition of sols onto monocrystalline sapphire substrates.

Due to different requirements of the employed characterization techniques we deposited and studied two sets of films. Each set was comprised of a film of carboxylated NPLs and a film of thiolated NPLs. Both sets were studied with UV-Vis absorption spectroscopy and XRF spectroscopy. Only the first set was studied with step-profilometry, whereas only the second set was studied with TXRF.

Spin-coating was performed on a custom-made apparatus. The rotation speed was 2270 rpm, the samples were deposited in 50 μL fractions in 3 s intervals. 0.7–1.1 mL of a sol were used to obtain a film. Typically, the volume used was greater for a sol of thiolated NPLs (the sol was perceived as more dilute due to decreased optical absorption, as will be discussed shortly).

### 2.6. Characterization and Equipment

The UV–Vis absorption spectra were recorded on Varian Cary 50 spectrometer (Varian Inc., Mulgrave, Victoria, Australia) against a clean sapphire substrate used as a baseline. IR spectra were taken on PerkinElmer Frontier FTIR spectrometer (PerkinElmer, Llantrisant, UK) with Pike diffusion reflection module. For the sake of this measurements, NPL sols were drop-cast on gold mirrors and dried.

Transmission electron microscopy (TEM) studies were conducted using a JEOL JEM-2100 microscope (JEOL Ltd., Tokyo, Japan) equipped with a LaB_6_ gun and an 11-megapixel Olympus Quemesa CCD camera (Olympus Soft Imaging Solutions GmbH, Münster, Germany). The accelerating voltage was set to 200 kV. Hexane sols containing NPLs were deposited onto carbon-coated copper grids and dried. The grids were then etched in argon plasma to remove organic material before examination. The sizes of the NPLs were measured from micrographs using Image-Pro Plus 6.0 software.

Composition analysis was conducted using XRF Bruker M1 Mistral spectrometer (Bruker Nano GmbH, Berlin, Germany) (tungsten anode) and a TXRF Bruker Picofox S2 spectrometer (Bruker Nano GmbH, Berlin, Germany) (MoKα radiation). In the case of XRF measurements the spectra were collected for 15 min. Several spectra were taken in different parts of the film with 2 mm radius of the film’s center. Constant intensity of the oncoming radiation was confirmed by recording the XRF spectra of a silver substrate prior to and after the measurements. In the case of TXRF studies, the outer regions of the spin-coat deposited film were removed until a roughly 3 mm in diameter spot was left in the center of the substrate. The beam width in the instrument is 1 cm.

The thickness of the films was determined with use of a Taylor-Hobson TalyStep profilometer (Taylor-Hobson Ltd., Leicester, UK). The films themselves are too soft to allow step-profilometry, so after grooves were made in a film, a thick (~0.5 μm) copper layer was deposited on top of it in a vacuum chamber of VUP-5 apparatus (Sumskii Zavod Elektronnikh Mikroskopov, Sumy, USSR) by thermal evaporation. The conditions for deposition were such that copper overcoating did not bias thickness determination (Section S1 of the Supplementary Information).

## 3. Results and Discussion

**Figure 1:**
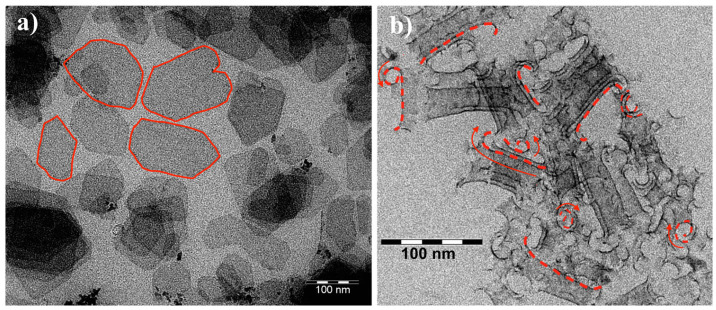
TEM images of: (**a**) carboxylated sample; (**b**) thiolated sample. Red dashed lines and arrows mark individual twisted fragments and indicate the rolling direction. Elongated outlines denote partially scrolled ribbons formed from free edges/corners of the original NPLs. The increased contrast arises from layer overlap.

We start our discussion by examining the dimensions and morphology of the studied particles. Determination of NPL thickness is a cumbersome task, however, it can be circumvented by the fact that it stays constant within a certain NPL population. The population itself is uniquely characterized by the position of its first absorption maximum. In our case, the position at 500 nm corresponds to 3.5 monolayer 1.9 ± 0.3 nm thick CdTe NPLs [18]. The lateral dimensions of carboxylated NPLs were obtained with TEM imaging (Figure 1a). The fabricated NPL gravitate towards predominantly rectangular shape, often with splayed angles. Their mean lateral dimensions are 110 × 70 nm^2^, the distributions of lengths, widths and basal plane areas are given in Figure S1 of the Supplementary Information. Exchange of stabilizer with thiol, expectedly [7], leads to formation of nanoscrolls with mean radius of curvature of 9 nm (Figure 1b).

**Figure 2:**
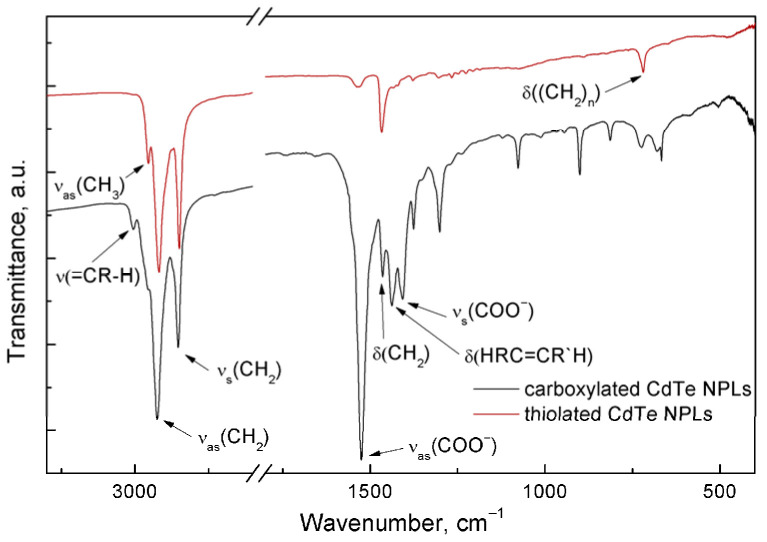
IR spectra of NPLs sample prior to thiol treatment (black line) and after thiol treatment (red line). The spectra are normalized for the intensity of valent C-H vibrations and offset vertically for clarity. The transmittance in the experiments was no less than 30% in the entirety of the spectrum, so the line shapes are not distorted. The break region in the plot is featureless.

Ligand exchange is confirmed by IR spectra as well (Figure 2). After thiol treatment almost the entirety of the spectrum is explained with C-H vibrations: functional group region only reveals characteristic C-H bands: *ν*_as_(CH_3_) at 2955 cm^−1^, *ν*_as_(CH_2_) at 2920 cm^−1^, *ν*_s_(CH_2_) at 2850 cm^−1^, scissoring vibration of CH_2_ at 1470 cm^−1^, rocking vibration of CH_2_ at 720 cm^−1^ [27]. At the same time, the contribution of carboxylate vibrations is greatly diminished: only the strongest band of *ν*_as_ can be seen at 1530 cm^−1^. The spectrum of the original NPLs is richer in features. For instance, ν(=C-H) is seen at 3010 cm^−1^ and *ν*_s_(COO^−^)—at 1410 cm^−1^ [9,27].

Next, we explore the properties of NPLs in the form of thin films. For these objects the question of thickness is of utmost importance. In this work we assessed the thickness with two methods: step-profilometry and XRF. Profilometry allows to study thickness at a granular level, assess the film’s surface roughness, measure absolute thickness values. Surface roughness was characterized with *Ra* parameter, the values of which were found for individual profiles and then averaged. The individual profile measurements for the film of thiolated NPLs were within 110–125 nm range and worked out to *Ra* of 120 nm. We averaged the thickness values within each profile and treated the result as a single measurement for the sake of statistics. The results are present in Table 1. The average thickness of this film is 590 ± 60 nm. The uniformity is about ±15% of this value within 2 mm radius of the film’s center—a region, from which TXRF and UV/Vis absorption data were collected and where the thickness was studied. The relative value of roughness of the film of thiolated NPLs amounts to 20%. For a film of carboxylated NPLs we likewise obtain *Ra* of 20 nm and a mean thickness of 127 ± 11 nm. The film is significantly thinner, but the quality in terms of relative parameters is similar to the thiolated case.

The thickness can also be evaluated via XRF, which serves as a complementary technique. When the thickness of the film is low, so that attenuation of outgoing characteristic X-rays is less than 5%, the intensity of characteristic fluorescence is proportionate to the areal density of an element. If we assume consistent density within one film and between different films of the same material and deposition conditions, then XRF intensity is proportionate to the film’s thickness. As tungsten anode bremsstrahlung maximum lies at lower energy than TeKα, we only computed the intensity of the CdKα line. We did so by integration in the spectral region of 22.96–23.36 keV after the background was fit locally with a straight line. The choice of integration window is to minimize the error (Figure S2 of the Supplementary Information). XRF measurements corroborate the profilometry results on film uniformity, the size of signal acquisition area does not allow to obtain roughness. We note here, that the precision of XRF as a thickness probe was comparable or better than that of step-profilometry, however, XRF thickness is in arbitrary units, so the final calculation would include the error of a direct method as well.

**Figure 3:**
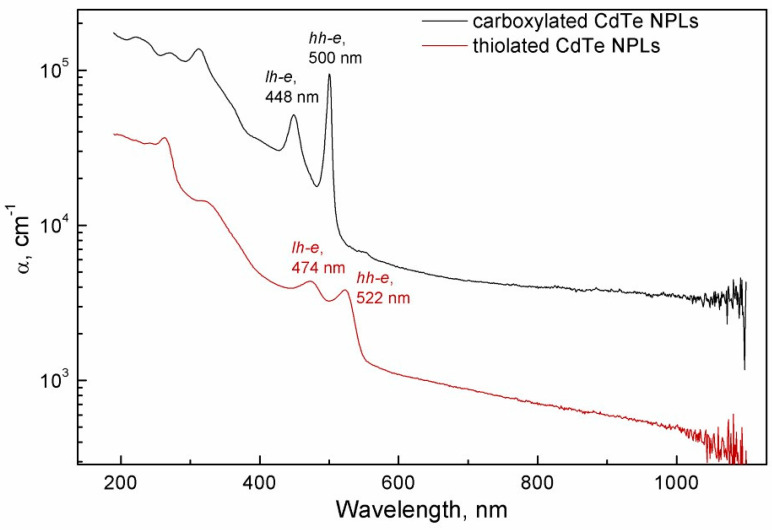
Spectral dependencies of absorption coefficients of films of carboxylated (black line) and thiol-exchanged (red line) NPLs.

As UV-Vis absorption spectroscopy is a non-destructive method, it was possible to study the same films prior to profilometry. Absorbance values can then be normalized by the thickness to produce the spectral dependencies of the absorption coefficient α (Figure 3).

A film made up of carboxylated NPLs attenuates light much more efficiently per unit length of optical path. In the region of 400–600 nm, where the respective first (electron-heavy hole) excitonic maxima of absorption are situated, the difference amounts to 1.5 orders of magnitude. Specifically, *α*_500_ for carboxylated NPLs (calculated as decadic absorbance *A*, divided by the film thickness *l*) is measured to be 94,700 cm^−1^, while *α*_523_ for the thiolated sample is 3840 cm^−1^. These can be immediately compared to the previously measured value of 1.5 × 10^4^ cm^−1^ for the films of the same carboxylated CdTe NPLs [18] and (2–3) × 10^4^ cm^−1^ measured for different populations of CdSe NPLs [15]. The observed differences between all these values are likely due to different efficiency of packing, as the films in Ref. [18] were obtained with drop-casting. For a crystalline CdTe slab, the absorption coefficient is around 10^4^–10^5^ cm^−1^ across the visible part of the spectrum [28]: the band edge is immediately in its vicinity in the IR region for this direct narrow-band semiconductor. Transition into ultradisperse state blue-shifts the band gap and increases the oscillator strength of HOMO-LUMO transition because of quantum confinement [29]. Taking all these considerations into account, our observations are consistent with dilute films of CdTe NPLs.

Absorption coefficient *α* is the product of molar absorption coefficient *ε* and concentration, which is directly proportional to density of the film with respect to the light-absorbing material. To decouple the contribution of these factors to the observed difference between carboxylated and thiolated NPL films, we have studied another set of films with TXRF and absorption spectroscopy.

So long as the film is infinitesimally thin for the characteristic fluorescence, absolute masses of the elements are determined within the signal acquisition spot. These can be normalized for the area of the film to obtain areal density. When the latter parameter is divided over the molar mass, we obtain the *c*·*l* product of the Beer-Lambert’s law. Selected parameters from TXRF studies are presented in Table 2. We have used the values of areal density to confirm the correctness of the analysis. Mass attenuation coefficients *μ* for CdLα, TeLα and SKα [30] were divided by cos(30°), which is the mean angle between the normal to the sample and the detector of our instrument, as discussed in another our work, which currently under consideration [31]. The attenuation by organic matter was introduced as an additional term to *μ*_Cd_ values, seen as organic stabilizers are bound to surface cadmium atoms. For the sake of this calculation, we estimated that 1 stabilizer moiety is present in the samples for every 2 Cd atoms (Cd_4_Te_3_X_2_ formula unit, where X is the organic stabilizer).

Even when multiplied by an angle-based factor, all *μ* values are below 1800 cm^2^/g (Table S1 of the Supplementary Information). From summation over the products *ρ_A_*·μ we obtain that every line in question would be attenuated by less than 1% even by the full thickness of the film. Mean attenuation is then less than 0.5%, which is lower than the uncertainly in area determination, so no correction for the sample self-absorption was adopted. The Cd-to-Te ratio has changed dramatically after the ligand exchange procedure. While there are no apparent indications of this process in TEM images, we suggest that some of the NPLs could be etched with the excess of hexadecanethiol. The resultant cadmium thiolate could adsorb on NPLs’ surface and therefore could remain unnoticed in the sample. Tellurides, on the other hand, are readily oxidized to the elemental form, which is insoluble and produces very apparent changes in the color of the sample. Preliminary Raman studies (532 nm excitation, Figure S3 of the Supplementary Information) confirm that CdTe phase is retained after ligand exchange. Due to that, we link the quantity of NPLs with Te content and normalize for it. Chalcogenide content has been used previously when there was a similar ambiguity in *ε* determination [14].

A factor of 4.63 ± 0.15 difference remains between the molar absorption coefficients. It follows that there is 5-fold change in the volume fraction of CdTe cores between the films of carboxylated and thiolated NPLs. This, of course, was indicated earlier by the XRF signal values for the first set of films (Table 1). We quantify the volume fraction by calculating concentration with the use of both *α* and *ε* values for the two sets of films, as well as density of bulk CdTe of 5.85 g/cm^3^. Films of carboxylated CdTe exhibit a volume fraction of inorganic cores of 8%, thiolated films—1.5%. The densest possible packing occurs through stacking of NPLs and interdigitation of organic terminating chains. In a stack on NPLs the volume fraction of cores can be assessed by relating the core thickness to the stacking period, measured with TEM. For the 500 nm population of alkylated CdTe NPLs an estimate of 30–40% can be obtained from the literature [18].

The difference in packing density appears feasible. Pre-formed scrolls are incapable of stacking in the same manner as flat NPLs. The folding is spontaneous and therefore should reach only local energy maxima. Inherent (comparatively) poor control over lateral dimensions of NPLs [32,33] leads to scrolls that are mismatched in their form, working further to slightly detriment the density of closest attainable packing. Moreover, there is a “dead volume” inside of the scroll, which is unable to accommodate other NPLs cores. We advocate for the combination of (T)XRF spectroscopy and profilometry of thin films as a fast and high-throughput technique to evaluate or control NPLs morphology. We have to note that XRF intensity is very well correlated to absorbance when we compare different films of the same material (Table 1 and Table 2, Figure 3).

When the difference in packing is accounted for, the difference in the intensity of optical response between NPLs with different anchor groups of ligands remains very substantial. Quantitative models for a quantum well always include direct proportionality between the absorption cross-section and frequency [14,16,18], however, between 500 and 523 nm there is only a factor of 1.05 difference. Next, we evaluate surface and dielectric effects. A particular study [34] has shown that the per-mole-value of ε for ZnSe colloidal quantum dots grows sharply with decreasing nanocrystal diameter, already reaching 10^4^ L·mol^−1^·cm^−1^ at the first maximum frequency for the mean diameter of 4 nm. This can be rationalized either with the effect of quantum confinement on the electron-hole pair, or with better electromagnetic field penetration of the cores [16]. Both these effects work to the advantage of NPLs in terms of the magnitude of ε [16,17], moreover, in the case of CdTe both elements are the heavier counterparts to the components of ZnSe, and have higher polarizability that is beneficial for light-matter interactions. The value of ε was measured at 2 × 10^4^ L·mol^−1^·cm^−1^ for 513 nm population of CdSe NPLs [14], which is in line with our findings and deductions. We note here, that the absorption efficiency is also frequently presented as intrinsic absorption coefficient, we calculate this value for oleate-capped CdTe NPLs to be 2.8 × 10^6^ cm^−1^ at the first exciton transition, even higher than that of 2 monolayer CdSe NPLs [17]. In the search for the ultimate light-absorbing material an investigation of thinner CdTe NPL populations is warranted.

The role of surface states was highlighted in the case of ZnSe quantum dots. Se-terminated cores have shown the values of ε at the maximum of first transition almost 2 times higher than that of their Cd-terminated counterparts [34]. In our case, what is essentially an addition of a layer of sulfur on the surface of NPLs lowers the molar absorption coefficient by about a factor of 5. To explain that we recall that in bulk the conduction band alignment of CdTe and CdS is rather close [35], and that strain can shift band energy in nanostructures to result in quasi-type II heterojunctions [36,37]. We believe that sulfur atoms in thiolated CdTe NPLs provide surface states for electrons thus decreasing the wavefunction overlap with holes.

## 4. Conclusions

We have constructed a robust protocol for determination of absorption coefficients of NPLs through addition of X-ray fluorescence spectroscopy to the existing group of characterization methods. Thin film form effectively combats the ever-present problem of NPL sedimentation and absorption on cuvette surfaces during the handling of sols. At the same time, minimal sample preparation required for the XRF studies eliminates possible associated systematic errors.

The molar absorption coefficient at the first optical transition of CdTe NPLs is measured at a significantly higher value (4.95 ± 0.12) × 10^4^ L·mol^−1^·cm^−1^ (per mole of Te) than in prior works, further proving the capacity of CdTe for light detection. When carboxylate stabilization is exchanged for thiolate this value drops to (1.07 ± 0.03) × 10^4^ L·mol^−1^·cm^−1^. This is additional evidence for formation of quasi-type II structure between CdTe cores and Cd thiolate capping layer.

Scroll-like morphology, attained through ligand exchange, translates to 5 times lower volume fraction of inorganic cores in the films fabricated by spin-coating. A combination of profilometry and XRF spectroscopy can then be employed to infer NPL morphology without TEM studies.

## Figures and Tables

**Table 1:** Thickness and XRF intensity for films of CdTe NPLs.

Sample	Carboxylated NPLs	Thiolated NPLs
Thickness, nm	113 ± 10	510 ± 70
	119 ± 15	520 ± 140
	120 ± 40	570 ± 80
	134 ± 18	610 ± 60
	135 ± 18	640 ± 130
	142 ± 15	690 ± 70
CdKα line intensity, a.u.	240 ± 30	200 ± 20
	230 ± 30	190 ± 20
	220 ± 30	190 ± 20
		190 ± 20
		200 ± 20
Absorbance	1.20	0.227

**Table 2:** Collated data on molar extinction coefficient determination for CdTe NPLs.

Sample		Carboxylated	Thiolated
*S*, cm^2^		0.0802 ± 0.0018	0.132 ± 0.003
*m*, ng	Cd	358 ± 3	626 ± 3
	Te	397 ± 3	404 ± 3
	S	1.8 ± 0.8	279 ± 3
Areal density, μg/cm^2^	Cd	4.46 ± 0.11	4.75 ± 0.11
	Te	4.95 ± 0.12	3.06 ± 0.07
	S	<0.04	2.12 ± 0.05
Stoichiometry parameter, *n*_Cd_/*n*_Te_		1.02	1.76
XRF intensity, a.u.		360 ± 30	190 ± 20
		370 ± 30	190 ± 20
		370 ± 30	190 ± 30
		372 ± 17	200 ± 30
			210 ± 20
Absorbance (wavelength, nm)		1.92 (500)	0.258 (523)
*ε* (per mole of Te), L·mol^−1^·cm^−1^		(4.95 ± 0.12) × 10^4^	(1.07 ± 0.03) × 10^4^

Please note that this study used a different set of films from the one presented in Table 1.

## Data Availability

The original contributions presented in the study are included in the article/Supplementary Materials; further inquiries can be directed to the corresponding authors.

## Supplementary Materials

The following supporting information can be downloaded at: https://www.mdpi.com/article/10.3390/nano15221688/s1, Section S1: Estimates for systematic error in step-profilometry due to copper overcoating, Figure S1: Histograms of linear dimensions of nanoplatelets under study, Figure S2: Relative error in CdKα line areal intensity determination as a function of integration interval, data for carboxylated NPLs film prepared for TXRF studies is used as an example, Table S1: Mass attenuation coefficients for self- and cross-absorption of nanoplatelet constituent elements, Figure S3: Raman spectrum of the thiolated NPLs film.
