# Supplementary material for: Observation of Direct and Indirect Effects of Surface Stabilizer on the Attenuation Coefficient of CdTe Nanoplatelet Films
Source: Nanomaterials (Basel). 2025 Nov 7;15(22):1688. doi: 10.3390/nano15221688 (PMC12655797; doi:10.3390/nano15221688)
Supplement: Supplementary file 1 [file nanomaterials-15-01688-s001.zip › nanomaterials-3935456-supplementary.pdf]

Supplementary Information for

Observation of Direct and Indirect Effects of Surface Stabilizer on the Attenuation Coefficient of CdTe Nanoplatelet Films

Sergei Bubenov 1,*, Aigerim Ospanova 2,*, Alexander Vinokurov 1, Asset Kainarbay 2, Aizhan Akhmetova 2, Kirill Cherednichenko 1, Dulat Daurenbekov 2,* and Sergey Dorofeev 1

[1] Department of Chemistry, Lomonosov Moscow State University, Leninskie Gory 1–3, 119991 Moscow, Russia

[2] Institute of Physical and Technical Sciences, L.N. Gumilyov Eurasian National University, Kazhymukan Str. 13, 010000 Astana, Kazakhstan

[*] Authors to whom correspondence should be addressed.

Section S1. Estimates for systematic error in step-profilometry due to copper overcoating

We envision two possible mechanisms of thickness determination bias due to copper overcoating.

The first one is through variable thickness of the copper layer. Metal was deposited from essentially a point source, perpendicular distance to the substrate a was 14 cm. The equation for the thickness of the film is the following:

$l = l_{max}\frac{a^3}{(a^2+r^2)^{3/2}}$; where l is the thickness, l_{max} is the maximum thickness, corresponding to the perpendicular from the source, r is the in-plane distance from perpendicular projection of the source onto the substrate.

We can estimate the changes in thickness on a small lateral scale (consistent with a step-profilometry experiment) with the use of a differential:

$$\mathrm{d}l = -3l_{max}\frac{a^3x}{(a^2+r^2)^{5/2}}\mathrm{d}r;$$

To evaluate the effect, we adopt an upper estimate of the in-plane distance of 3 cm, dr value of 2 mm, corresponding to the maximum possible measurement length on our device, l_{max} of 0.5 µm. The upper estimate of the thickness change in a single measurement is then calculated to be as low as 1.4 nm, much lower than the error margins reported.

The second mechanism is obstruction of deposition inside the groove by the film edges, that can occur when the origin is not aligned perfectly normal to the groove. The grooves in our case were quite wide: about 40-100 micrometers, their width is at least 2 orders of magnitude greater than thickness of the films. Due to that, the impact of the half-shadow of the film’s edge is negligible.

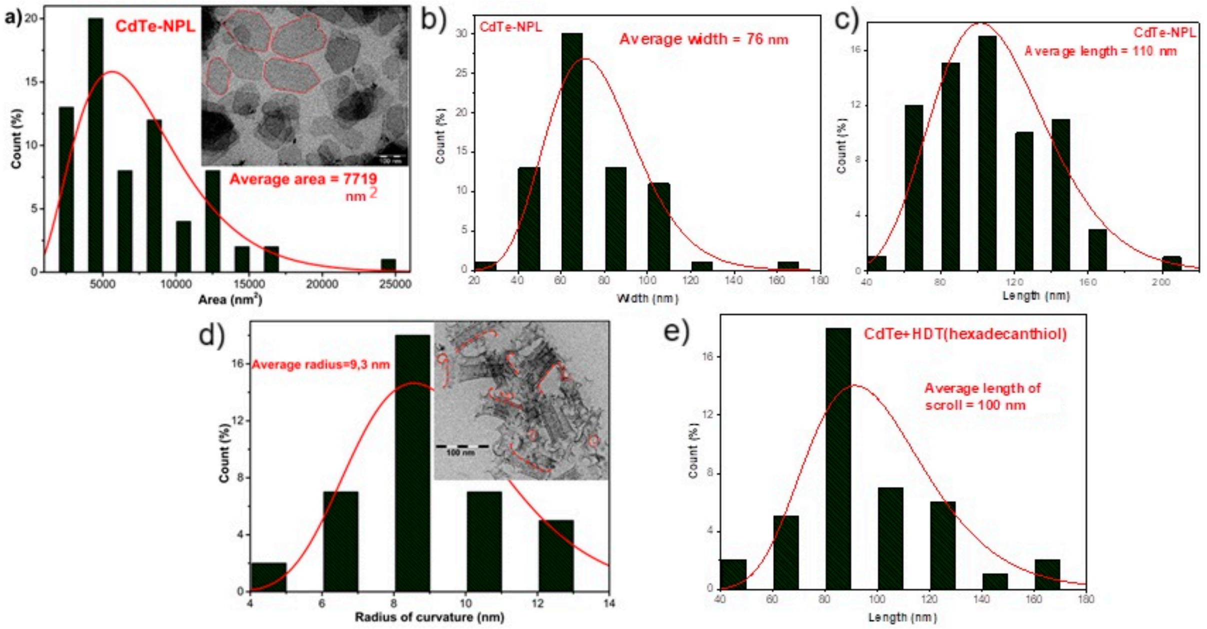

Figure S1. Histograms of linear dimensions of nanoplatelets under study: a) area of oleate-covered CdTe; b) width of oleate-covered CdTe; c) length of oleate-covered CdTe; d) radius of curvature of nanoscrolls of thiol-covered CdTe; e) scrolling axis length of nanoscrolls of thiol-covered CdTe.

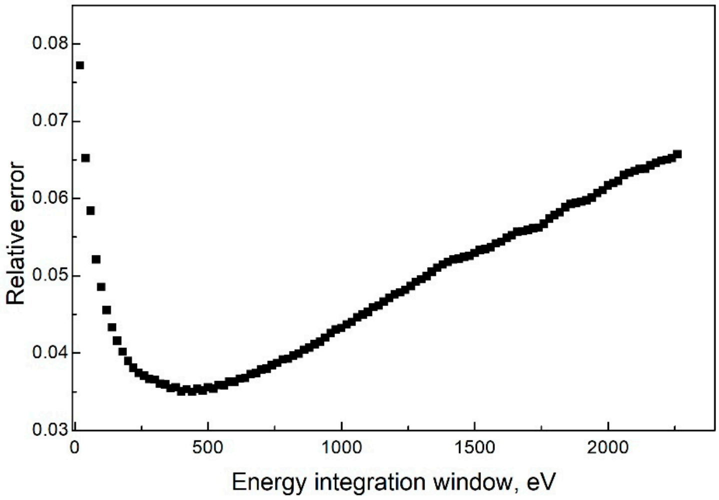

Figure S2. Relative error in CdKα line areal intensity determination as a function of integration interval, data for carboxylated NPLs film prepared for TXRF studies is used as an example.

Table S1. Mass attenuation coefficients for self- and cross-absorption of nanoplatelet constituent elements. Single asterisk denotes addition of attenuation of a half of oleate moiety, double asterisk – a half of hexadecanethiolate moiety. Angle adjusted values incorporate longer optical path that is the result of acquisition of signal by detector over a large solid angle.

Line	Mass attenuation coefficient, cm^2/g										
	Reference [1]					effective		angle-adjusted			
	Cd	Te	S	C	O	Cd*	Cd**	Cd*	Cd**	Te	S
CdLα	534	688	1240	73.3	177	630	597	727	689	795	1432
TeLα	1425	440	740	42.8	103	1481	1462	1710	1688	508	855
SKα	1130	1490	237	179	433	1575	1283	1575	1482	1721	274

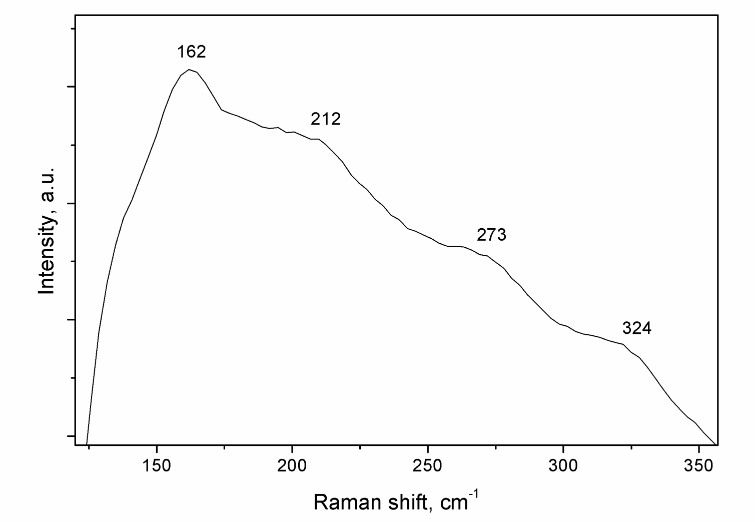

Figure S3. Raman spectrum of the thiolated NPLs film. The spectrum was measured with a iRamanPlus portable spectrometer (532 nm excitation), the integration time was 1 second.
